# Prefrontal Cortex Activity Is Associated with Biobehavioral Components of the Stress Response

**DOI:** 10.3389/fnhum.2016.00583

**Published:** 2016-11-17

**Authors:** Muriah D. Wheelock, Nathaniel G. Harnett, Kimberly H. Wood, Tyler R. Orem, Douglas A. Granger, Sylvie Mrug, David C. Knight

**Affiliations:** ^1^Department of Psychology, University of Alabama at Birmingham, BirminghamAL, USA; ^2^Institute for Interdisciplinary Salivary Bioscience Research, University of California at Irvine, IrvineCA, USA; ^3^Johns Hopkins University School of Nursing, Johns Hopkins University Bloomberg School of Public Health, and Johns Hopkins University School of Medicine, BaltimoreMD, USA

**Keywords:** anxiety, stress, fMRI, cortisol, PFC, MIST

## Abstract

Contemporary theory suggests that prefrontal cortex (PFC) function is associated with individual variability in the psychobiology of the stress response. Advancing our understanding of this complex biobehavioral pathway has potential to provide insight into processes that determine individual differences in stress susceptibility. The present study used functional magnetic resonance imaging to examine brain activity during a variation of the Montreal Imaging Stress Task (MIST) in 53 young adults. Salivary cortisol was assessed as an index of the stress response, trait anxiety was assessed as an index of an individual’s disposition toward negative affectivity, and self-reported stress was assessed as an index of an individual’s subjective psychological experience. Heart rate and skin conductance responses were also assessed as additional measures of physiological reactivity. Dorsomedial PFC, dorsolateral PFC, and inferior parietal lobule demonstrated differential activity during the MIST. Further, differences in salivary cortisol reactivity to the MIST were associated with ventromedial PFC and posterior cingulate activity, while trait anxiety and self-reported stress were associated with dorsomedial and ventromedial PFC activity, respectively. These findings underscore that PFC activity regulates behavioral and psychobiological components of the stress response.

## Introduction

The biobehavioral response to acute stress is typically considered an allostatic process (McEwen and Wingfield, 2003; Karatsoreos and McEwen, 2011). However, dysregulation of the stress response has been implicated in allostatic load and the pathophysiology of a wide range of disorders (McEwen and Gianaros, 2011). Despite considerable advances within the last two decades investigating the correlates of allostatic load (McEwen and Gianaros, 2011), a consistent finding across studies is that individual differences are the norm rather than the exception. When individuals confront adverse circumstances early in life, intrinsic individual differences in biological sensitivities or susceptibilities have the capacity to translate those experiences into health-related disparities later in life (McEwen, 2008; Danese and McEwen, 2012). Determining the neural processes associated with individual variability in the stress response has the potential to advance our understanding of the nature of individual differences and stress-related disorders.

The hypothalamic-pituitary-adrenal (HPA) axis is an important component of the psychobiological response to stress (Herman et al., 2005). HPA axis activity can be measured non-invasively using salivary cortisol (Granger et al., 2012), and large inter-individual variability in the activity of the HPA axis is a well-documented phenomenon in psycho-neuroendocrine studies (Kirschbaum et al., 1993; Negrao et al., 2000; Kudielka et al., 2009). Prefrontal cortex (PFC) activity is known to influence activity in the hypothalamus and amygdala (Dedovic et al., 2009a), and therefore the PFC may directly, or indirectly, influence the individual variability observed in HPA axis reactivity to stress.

Prefrontal cortex activity may be associated with several facets of the psychological and physiological response to stress. For example, prior research has demonstrated trait anxiety varies negatively with ventromedial PFC (vmPFC) activity and positively with dorsolateral (dlPFC) and dorsomedial PFC (dmPFC) activity during psychological tasks (Indovina et al., 2011; Basten et al., 2012; Wood et al., 2012). Similarly, greater self-reported stress elicited by a psychological stressor has been linked to greater neural activity within the vmPFC and dmPFC (Wang et al., 2005). Finally, prior stress studies using psychological and psychosocial stressors have demonstrated that cortisol release is positively associated with activity in the dmPFC (Wang et al., 2005; Dedovic et al., 2009b). However, the breadth of prior work linking cortisol release and vmPFC activity is mixed, with some studies finding a positive association between cortisol and vmPFC activity (Wang et al., 2005; Jahn et al., 2010), and others observing a negative correlation between cortisol and activity in the vmPFC (Urry et al., 2006; Kern et al., 2008; Pruessner et al., 2008; Albert et al., 2015). Interestingly, several studies have observed no correlation between trait anxiety, self-reported stress, and cortisol reactivity (Bohen et al., 1991; Vedhara et al., 2003; Takai et al., 2004; Takahashi et al., 2005; Albert et al., 2015; Chung et al., 2016) which suggests that self-reported stress, trait anxiety, and cortisol assess distinct stress-related processes. Few studies to date have assessed trait anxiety, self-reported stress, and cortisol response within the same set of participants (Wang et al., 2005). While prior studies suggest the PFC influences these three biobehavioral correlates of the stress response, no prior research has directly investigated the overlap in PFC function associated with these measures.

The present study investigated whether the neural response to stress is associated with trait anxiety (State-Trait Anxiety Inventory; STAI), self-reported stress, and cortisol release. Functional magnetic resonance images (fMRI) were acquired to measure the neural response to stress using a variation of the Montreal Imaging Stress Task (MIST) (Dedovic et al., 2005). Heart rate (HR) and skin conductance responses (SCR) were measured concurrently with fMRI as additional physiological indices of stress. Salivary cortisol was assessed prior to and following the MIST. We expected variability in trait anxiety (Wang et al., 2005; Wood et al., 2012), self-reported stress (Wang et al., 2005), and cortisol reactivity (Pruessner et al., 2008; Dedovic et al., 2009b) to vary with brain activity within the vmPFC and dmPFC. Further, we investigated whether the activity associated with anxiety, self-reported stress, and cortisol co-localized within the PFC.

## Materials and Methods

### Participants

Fifty-three right-handed volunteers (30 males, 23 females, mean age = 18.68, *SEM* = 0.13, age range 17–22 years, 30 African American, 23 Caucasian) participated in an fMRI study using a variation of the MIST (Dedovic et al., 2005). Exclusion criteria consisted of seizure history, disease affecting blood flow (sickle cell, diabetes, etc.), or prior loss of consciousness. All participants provided written informed consent as approved by the University of Alabama at Birmingham Institutional Review Board.

### Task Design

Participants completed a modified version of the MIST, a challenging mental arithmetic task optimized for administration during fMRI (Dedovic et al., 2005). This study used a fast event-related design consisting of two scans (Control MIST followed by Stress MIST). Each scan was 7 min and 54 s in duration and contained 54 trials. Each trial (**Supplementary Figure S1**) was separated by a 1–3 s inter-trial interval (fixation cross) and lasted 6 s. Trials consisted of a unique math problem that coincided with a window of time for participant response (0.5–5 s duration) followed by a fixation cross (0.5–5 s duration) and then 0.5 s of visual feedback (“Right,” “Wrong,” or “Time out”). The presentation of each math problem coincided with the presentation of possible answers ranging from 0 to 9 (**Supplementary Figure S1**). Each scan ended with a 14 s presentation of the fixation cross. The order of Control and Stress MIST was not counterbalanced to (1) avoid the stress carry over effects that might develop when the stress condition precedes the control condition; (2) reduce variability induced by counterbalancing order to better isolate differences related to inter-subject variability in our measure of interest.

Prior to the scanning session, participants completed a set of practice math problems outside of the scanning environment to familiarize them with the task and determine the difficulty level of problems to be presented during scanning. Participants answered math problems ranging in difficulty level from easy (addition or subtraction of two single digit integers), medium-easy (addition or subtraction of three single digit integers), medium-hard (addition or subtraction of four single or double digit integers), and hard (addition, subtraction, and multiplication of four single or double digit integers) problems during practice. No participants were able to reliably complete medium-hard or hard practice problems in the allotted time (i.e., less than 5 s) during practice. Therefore, all participants received either easy or medium-easy math problems in the scanner based on their response time during the practice session. The difficulty level (easy or medium-easy math problems) remained constant across Control and Stress scans.

Prior to the Control scan, investigators attempted to lower participant stress levels by telling them “It is OK if you do not answer all of the math problems correctly.” During the Control scan the participants were given 5 s in which to respond to each math problem. Further, during the Control scan, participants were given previously recorded positive auditory feedback. For example, phrases such as “Good job, you’re doing just fine. Keep up the good work.” and “Looks like everything is going well. You’re doing just what we wanted, so keep it up.” were presented. The Stress scan began approximately 1 min after the Control Scan ended, with just enough time for the investigators to read a script intended to elevate participant stress levels. Investigators informed the participants they must answer the questions correctly, and warned that if they did not perform as well as others in the study their data would not be used. In addition, participants were told that prior subjects answered more than 80% of the answers correctly, and if he/she did not answer at least 80% correct his/her data would not be used. Further, during the Stress scan, the participants were given recorded negative auditory feedback. For example, phrases such as “You are not answering enough of these correctly. Please try as hard as you can to get these right.” and “You are not doing as well as we had hoped. Please do your best to answer these correctly.” were presented. Failure was ensured by modulating the time in which the participant could respond in a stair-step manner such that on average participants answered approximately 50% of the problems correctly. The stair-step procedure was implemented by decreasing the available time by 0.5 s for each correct answer, and increasing the time by 0.5 s for each incorrect answer. Thus, the time available to answer math problems could vary between 0.5 s (minimum) and 5.0 s (maximum) in 0.5 s increments. Once a math response was selected (or response time expired) a fixation cross (0.5–5 s duration) appeared on the screen until the trial ended with 0.5 s of visual feedback (“Right,” “Wrong,” or “Time out”).

### Task Presentation

Presentation software (Neurobehavioral Systems, Inc.; Albany, CA, USA) was used to present the visual stimuli through an IFIS-SA LCD (Invivo Corporation; Gainesville, FL, USA) video screen located above the participant’s head. The participants were able to view the video screen through a mirror attached to the RF coil. Participants used an MRI compatible joystick (Current Designs; Philadelphia, PA, USA) to highlight their math answer in yellow (**Supplementary Figure S1**) and a button on the joystick to make their selection. Participants’ responses to the math problems were used to provide corresponding real time visual feedback on task performance (e.g., ‘Right,’ ‘Wrong,’ or ‘Time out’). Prerecorded auditory feedback was presented at four fixed points (i.e., after the first four sets of nine trials) during each scan through MR-compatible pneumatic headphones.

### Trait Anxiety

Participants completed the State-Trait Anxiety Inventory (STAI form Y, Spielberger, 1983) prior to the imaging session. Scores on the trait anxiety scale were assessed as an index of participants’ general tendency to engage in negative affect, and used for comparison to the neural response to stress. State anxiety scale scores were not compared with brain activity.

### Self-Reported Stress

A measure of self-reported stress level was developed as a manipulation check of participant’s emotional response to Control and Stress MIST scans. Following the completion of the MIST, participants completed a self-report questionnaire consisting of eight statements. Participants rated each statement’s applicability on a five point scale where 1 corresponded to “not at all” and 5 corresponded to “Extremely.” Four of the statements were worded positively (e.g., I felt calm) and four were worded negatively (e.g., I felt stressed) for a total possible self-reported stress score of 40.

### Math Performance

Math task performance was assessed as a manipulation check to confirm that task performance varied between Control and Stress MIST scans as designed. While the difficulty of math problems remained constant for each participant, the response time window was titrated during Stress MIST to obtain an approximately 50% performance level. Therefore, math task performance was calculated as the percentage of correct answers during Control and Stress MIST.

### Skin Conductance Response

Skin conductance response data were collected using an MRI compatible physiological monitoring system (Biopac Systems; Goleta, CA, USA) using the basic methodology described in prior work (Knight and Wood, 2011). SCR was sampled at 10 kHz with a pair of disposable radio-translucent electrodes (1 mm diameter, Biopac Systems; Goleta, CA, USA) located on the thenar and hypothenar eminence of the non-dominant hand. SCR data were low pass filtered at 1 Hz and downsampled to 250 Hz using Acqknowledge 4.1.0 software. The downsampled SCR were analyzed with SCRalyze toolbox (version b2.1.8) (Bach et al., 2009). The data were then bandpass filtered with a first order Butterworth filter (highpass cutoff of 0.0159 Hz, lowpass filter of 1.0 Hz) and further downsampled to 10 Hz sampling rate. The time-series was normalized (z-transformed and mean centered). SCRs to math events were estimated using the general linear model with an assumed SCR function without a time or dispersion derivative.

### Heart Rate

Heart rate was collected using an MR compatible photoplethysmograph placed on the index finger of the non-dominant hand. HR was recorded at 50 Hz using a Siemens Physiological Monitoring Unit. QRSTool (Allen et al., 2007) was used to identify peaks in the pulse waveform. CMetX (Allen et al., 2007) was used to calculate the average HR for Stress and Control scans.

### Cortisol Collection

Two saliva samples were collected to assess the cortisol response to the MIST. The first sample (Time 1) was collected 30 min after participant arrival, just prior to the scanning session. The second sample (Time 2) was collected 20 min following the MIST. A third sample (Time 3) was collected 60 min after the MIST following a Pavlovian fear conditioning task described elsewhere (Harnett et al., 2015), but was not included in the present analysis. Salivary cortisol samples (1.0 ml) were collected using passive drool through a short straw into 2.0 ml cryovials, then stored at -80°C. Prior studies have sampled cortisol only during the afternoon to control for diurnal rhythms. However, due to limitations in participant availability, data collection was completed between the hours of 9 am to 5 pm. Thus, there was variability in the recorded time of first saliva sample collection. Approximately 30% of salivary data was collected before 12 pm (Mean = 13:18 h, *SEM* = 0.34 h, Range 0900–1700 h).

### Cortisol Analysis

Samples were shipped overnight on dry-ice for assay at the Institute for Interdisciplinary Salivary Bioscience Research (Arizona State University). On the day of testing, all samples were thawed and centrifuged at 3,000 rpm for 15 min to remove mucins. Samples were assayed for cortisol using the cortisol enzyme immunoassay kit (Salimetrics, LLC in State College, PA, USA) following the manufacturers recommended protocol. The cortisol assay used 25 μl of saliva for singlet determinations and had a range of sensitivity from 0.007 to 3 μg/dl. Samples were assayed in duplicate and the average of the duplicate assays were used in the statistical analyses. On average, intra- and inter-assay coefficients of variation were less than 10 and 15%. Cortisol data were transformed to nmol/L and cortisol outliers (3 SD from the mean) were assessed and winsorized at the 97th percentile prior to statistical analyses.

The effect of MIST on baseline (Time 1) to post-stress (Time 2) cortisol levels was assessed for the whole group using a 1-way repeated measures ANCOVA (including time of day as a covariate). Similar to previous MIST studies (Pruessner et al., 2008; Dedovic et al., 2009b), intersubject variability was expected in the cortisol response to the MIST. To estimate cortisol reactivity post-MIST cortisol data (Time 2) were regressed on pre-MIST cortisol (Time 1) and time of day of first saliva collection. The residual from this regression analysis was then used to determine individuals with high (Responders) vs. low (Non-responders) cortisol reactivity from pre-MIST to post-MIST. Furthermore, intersubject variability in raw cortisol and cortisol residual score attributable to behavioral measures including trait anxiety, self-reported stress ratings, HR, and SCR was assessed using Pearson correlations.

### Functional MRI

Blood oxygen level dependent (BOLD) fMRI was acquired on a 3T Siemens Allegra Scanner using a brain-specific single channel RF head coil. Functional MRI data were acquired using a gradient-echo pulse (EPI) sequence (TR = 2000 ms, TE = 30 ms, FOV = 24 cm, matrix = 64 × 64, slice thickness = 4 mm) during two scans of stimulus presentations. High resolution anatomical images (MPRAGE) were also obtained to serve as an anatomical reference (T1 weighted, TR = 2300 ms, TE = 3.9 ms, FOV = 25.6 cm, matrix = 256 × 256, 160 slices, slice thickness = 1 mm, 0.5 mm gap). MRI data were preprocessed using the AFNI software package (Cox, 1996). Functional MRI data were slice time corrected, spatially blurred using a 4 mm full-width-at-half-maximum Gaussian kernel, and coregistered to the MPRAGE. Functional MRI data were corrected for motion by censoring high-motion TRs, and including the six motion parameters in the first level model. High motion TRs were defined as volumes in which greater than three percent of voxels deviated by more than five times the median absolute signal. Participants with less than 80% usable TRs were excluded from data analysis. First level models included a regressor of interest for math problems, as well as regressors of no interest for auditory feedback events, visual feedback events, joystick movement, button presses, and the six head motion parameters. Functional MRI data were regressed against events of interest using the gamma variate hemodynamic response function. Visual feedback events were modeled using an instantaneous response function while math task and audio feedback events were modulated using the duration of the response time (math problem onset to answer selection or time out) and the duration of the audio recording, respectively. Functional MRI data from math events were resampled to 1 mm^3^ and normalized to the MNI 152 template.

### Brain and Behavior Analyses

Group level analyses were completed using paired samples *t*-tests (3dttest++ in AFNI) to compare Stress vs. Control task-elicited brain activity. Trait anxiety score and self-reported stress were regressed on Stress vs. Control brain activation (using 3dttest++). Because self-reported stress data were not collected on the first 13 subjects, trait anxiety, self-reported stress, and cortisol Responder vs. Non-responder labeling were not included within the same regression. Responder vs. Non-responder analyses of brain activation were performed using independent samples *t*-test (3dttest++). Analyses were restricted to a gray matter mask to reduce the number of voxel-wise comparisons in the analysis. Data were cluster corrected using 3dClustSim at a family wise error rate of *p* < 0.05. Further, given findings from prior research (Wang et al., 2005; Pruessner et al., 2008; Dedovic et al., 2009b; Indovina et al., 2011; Basten et al., 2012; Wood et al., 2012), we were particularly interested in identifying areas of overlap among these three metrics rather than partialling variance among them. Therefore, the commonalities in activation between Trait anxiety, self-reported stress, and cortisol response were assessed using a conjunction analysis. To assess the conjunction of common activation across these three contrasts, activation results from each contrast were thresheld at the minimum *t*-value (identified by 3dClustSim), turned into masks, and multiplied together. The conjunction masks were then thresheld at a voxel extent of 3000 voxels (1 mm × 1 mm × 1 mm voxel size) (the minimum cluster extent identified in 3dclustsim). Only areas of common activation surpassing this *t*-value and cluster extent threshold were included in the results, thus correcting the conjunction analysis for family wise error rate of *p* < 0.05.

## Results

### State and Trait Anxiety

The State (Mean = 33.92, *SEM* = 1.22, Range = 20–53) and Trait (Mean = 35.47, *SEM* = 1.22, Range = 20–55) anxiety inventory (Spielberger, 1983) was completed as an index of negative affect prior to the scanning session. State and Trait anxiety inventory scores were correlated (*r* = 0.454, *p* < 0.001). Trait anxiety was used as an index of general negative affect in comparisons with behavioral and neuroimaging data.

### Self-Reported Stress

**FIGURE 1:**
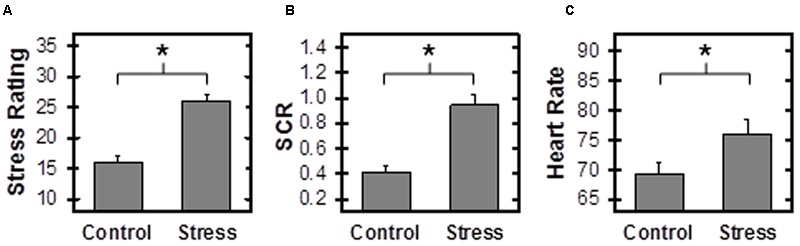
**Stress Response.** Self-reported stress **(A)**, skin conductance response (SCR) **(B)**, and heart rate (HR) **(C)** were higher during Stress than Control Montreal Imaging Stress Task (MIST) conditions. SCR, Skin Conductance Response, calculated as the beta estimate of the SCRs to Math events. ^∗^Reflects *p* < 0.05.

Ratings of self-reported stress were obtained for Control and Stress conditions of the MIST as a manipulation check. Self-reported stress was not collected for first 13 participants. Thus, self-reported stress for these participants were not included in analyses of these data. Paired *t*-test comparisons of self-reported stress indicate that participants found the Stress condition more stressful than the Control condition (**Figure 1A**; **Table 1**).

**Table 1:** Paired samples *t*-tests comparing Control to Stress Montreal Imaging Stress Task (MIST).

Measure	Control (*M* ±*SEM*)	Stress (*M* ±*SEM*)	*n*	*t*	*p*
Response time	2.91 ± 0.02	2.21 ± 0.02	53	17.88	<0.001
% Correct responses	85% ± 1%	54% ± 0.1%	53	23.06	<0.001
SCR	0.42 ± 0.05	0.94 ± 0.08	44	-7.34	<0.001
Stress rating	15.55 ± 0.59	25.98 ± 0.59	40	-8.82	<0.001
HR	69.38 ± 5.54	76.06 ± 5.54	40	-5.15	<0.001

*Response time (in seconds) indicates time following math problem presentation to response selection with a button press. HR, Heart Rate; SCR, Skin Conductance Response. Stress ratings were not collected on 13 participants. Seven participants had errors in HR data acquisition, and six participants had HR with low SNR. One participant had errors in SCR data acquisition, and 10 did not have any SCRs above 0.05 μSiemens. SEM reflects within subject standard error of the mean.*

### Skin Conductance Response

Skin conductance response was monitored during the scanning session as an index of the peripheral emotional response to Stress and Control MIST. One participant did not have SCR data due to equipment failure and ten individuals had no measurable SCRs (i.e., zero SCRs above 0.05 μSiemens). Thus SCR data were assessed for 44 participants. Paired *t*-test comparisons revealed that SCRs to math events were significantly greater during Stress than Control conditions (**Figure 1B**; **Table 1**). These data demonstrate differential SCR to Stress vs. Control scans, and provide behavioral evidence the Stress condition was more stressful than the Control condition. SCR did not vary with HR, cortisol, self-reported stress, or trait anxiety scale score.

### Heart Rate

Heart rate was also monitored during the scanning session to assess the differential emotional response to Stress and Control MIST. HR data were not collected from seven subjects due to equipment malfunction. In addition, HR could not be calculated for six participants due to excessive noise in the signal. Paired *t*-test comparisons indicate HR was significantly higher during Stress than Control MIST (**Figure 1C**; **Table 1**). These data demonstrate differential cardiac response to Stress vs. Control scans, and provided additional behavioral evidence the Stress condition was more stressful than the Control condition. HR did not vary with SCR, cortisol, self-reported stress, or trait anxiety scale score.

### Math Performance

The percentage of correctly answered items during Stress and Control scans was calculated as a manipulation check. During the Control scan, participants answered 85.85% correct (range 57–100, *SEM* = 0.02), whereas participants only answered 54.05% correct (range 44–57, *SEM* = 0.03) on the Stress scan (**Table 1**). These findings confirm that performance varied across scans as designed.

### Cortisol

Cortisol data from one participant could not be analyzed due to the poor quality of the sample. Cortisol values were positively skewed, with one outlier (greater than 3 SD above the mean). Therefore, raw cortisol scores were winsorized at the 97th percentile. Raw cortisol at Times 1 and 2 was not correlated with age, state anxiety, trait anxiety, or stress rating, and did not differ between sexes or ethnicities (**Table 2**). Cortisol values were negatively correlated with time of day at Time 1 (*r* = -0.62, *p* < 0.001) and Time 2 (*r* = -0.57, *p* < 0.001).

**Table 2:** Relationship between raw cortisol and other metrics.

Measure	Time 1 Cortisol		Time 2 Cortisol	
	*r*	*p*	*r*	*p*
Gender	-0.228	0.104	-0.095	0.503
Race	0.214	0.127	0.115	0.413
Age	-0.153	0.279	0.028	0.851
State anxiety	0.056	0.695	0.207	0.142
Trait anxiety	0.082	0.565	0.001	0.995

*All *p* > 0.05.*

### Cortisol Responders

**FIGURE 2:**
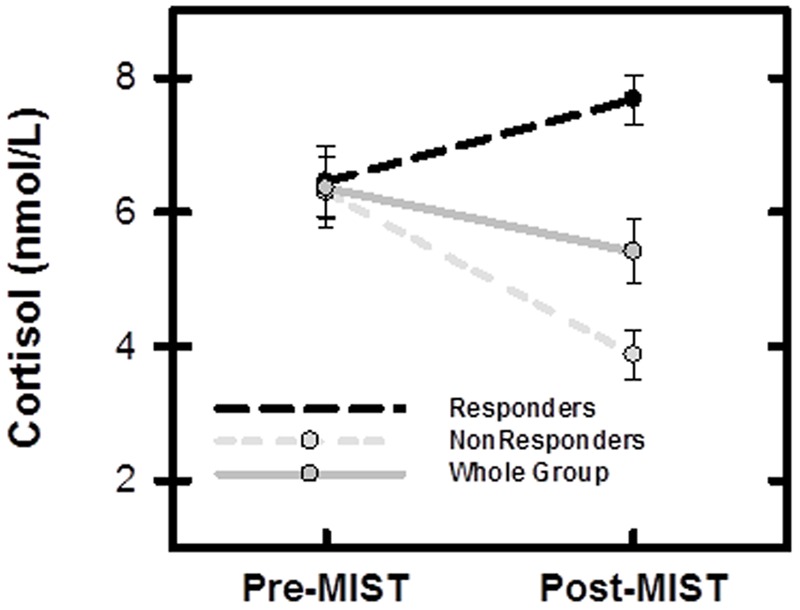
**Salivary cortisol measurement (mean and SEM) for entire sample (*N* = 52), Responders (*n* = 22), and Non-responders (*n* = 30).** Pre-MIST: immediately prior to scanning session, Post-MIST: 20 min after end of MIST.

Whole group cortisol levels did not increase after the MIST [*F*(1,50) = 0.26, *p* > 0.05] (**Figure 2**). However, as observed in prior MIST papers (Pruessner et al., 2008; Dedovic et al., 2009b), a heterogeneous cortisol response was observed in which some participants showed an increase in cortisol level post-Stress while others showed a decrease in cortisol post-Stress. Therefore, *post hoc* exploratory analyses were performed in which participants were split into two groups of cortisol ‘Responders’ and cortisol ‘Non-responders,’ using a linear regression in which post-MIST cortisol (Time 2) was regressed on pre-MIST cortisol (Time 1) and time of day of sample collection (time). Cortisol ‘Responders’ were identified as those individuals with positive residual cortisol values and ‘Non-responders’ were individuals with negative residual cortisol values. This residual split resulted in 22 ‘Responders’ and 30 ‘Non-responders’ (**Figure 2**). A two factor mixed design ANOVA revealed a significant group (i.e., Responder vs. Non-responder) × time (Time 1 vs. Time 2) interaction [*F*(1,49) = 42.65, *p* < 0.001]. *Post hoc* tests revealed that Responders showed a significant increase in cortisol level after the MIST [*t*(20) = 2.30, *p* < 0.05], whereas Non-responders showed a significant decrease in cortisol [*t*(30) = -8.91, *p* < 0.001]. Similar to prior MIST research (Pruessner et al., 2008; Dedovic et al., 2009b), cortisol Responder and Non-responder groups were used to investigate the neuroendocrine response to stress. Cortisol Responders and Non-responders did not differ in age, gender, ethnicity, trait anxiety, state anxiety, self-reported stress, math problem difficulty level, number of correct responses, time of data collection, response time, or SCR (**Table 3**). No correlations were observed between trait anxiety, cortisol reactivity, self-reported stress, SCR, and HR within the Cortisol Responder or Non-responder groups.

**Table 3:** Cortisol Responders (*n* = 21) compared to Non-responders (*n* = 31).

Measure	Non-responders (*M* ±*SEM*)	Responders (*M* ±*SEM*)	*n*	*t/x*^2^	*p*
Task difficulty	1.23 ± 0.08	1.36 ± 0.11	52	1.015	0.315
State anxiety	32.17 ± 1.42	36.00 ± 2.12	52	1.315	0.195
Trait anxiety	35.17 ± 1.76	36.05 ± 1.73	52	0.346	0.732
Gender	17 M, 14 F	12 M, 9 F	52	0.027	0.870
Race	12 C, 19 AA	11 C,10 AA	52	0.949	0.330
Age	18.60 ± 0.16	18.86 ± 0.22	52	0.981	0.331
Time of day	13.47 ± 0.41	13.07 ± 0.58	52	-0.577	0.566
Response time	-0.68 ± 0.05	-0.71 ± 0.07	52	0.455	0.651
% correct	-0.32 ± 0.02	-0.032 ± 0.02	52	-0.006	0.995
SCR	0.55 ± 0.10	0.47 ± 0.12	43	0.588	0.560
Stress rating	10.00 ± 1.65	10.85 ± 1.73	40	-0.355	0.724
HR	5.00 ± 1.10	9.48 ± 2.69	39	-1.721	0.094

*Response time, % correct, skin conductance response (SCR), stress rating, and heart rate (HR) are reported as differential values (Stress-Control). Response time (difference in seconds between Stress and Control) indicates time following math problem presentation to response selection with a button press. M, Male; F, Female; C, Caucasian; AA, African American.*

### Functional MRI

**FIGURE 3:**
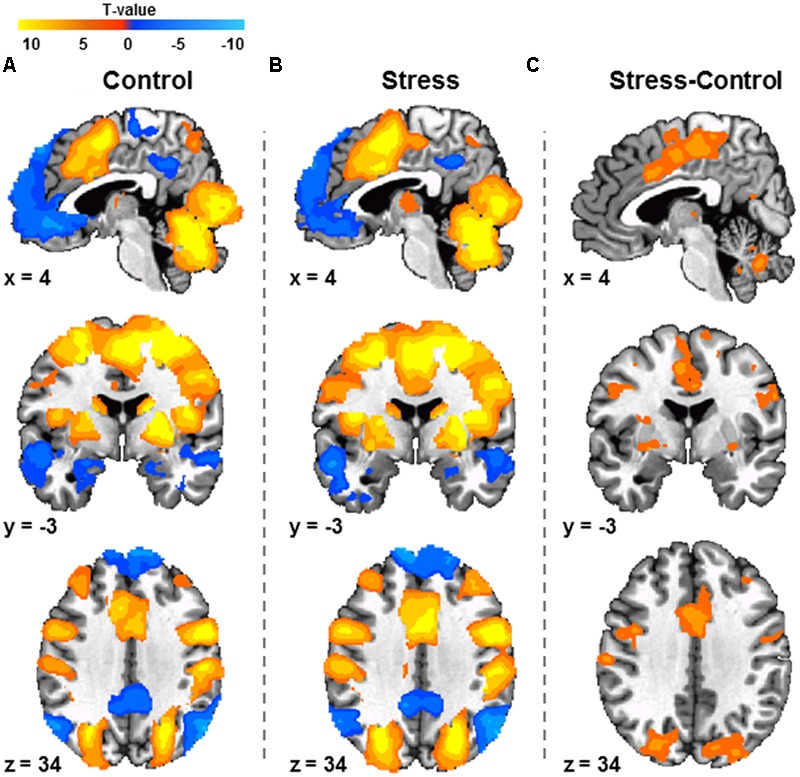
**Response to Control MIST** **(A)**, Stress MIST **(B)**, and the differential (Stress vs. Control) response during the MIST **(C)** (*N* = 53). Differences were observed between Stress and Control conditions within regions including the dorsomedial prefrontal cortex, dorsolateral prefrontal cortex, and inferior parietal lobule. Images are FWE corrected *p* < 0.05 and presented in radiologic view.

A paired sample *t*-test revealed greater differential activity during Stress than Control conditions of the MIST across several cortical and sub-cortical structures (**Figure 3**; **Supplementary Table S1**). Specifically, greater differential activity was observed within the dmPFC, dlPFC, and parietal cortex. No regions were found to be more activated during Control than Stress conditions.

### Cortisol Responder and Non-responder Brain Results

**FIGURE 4:**
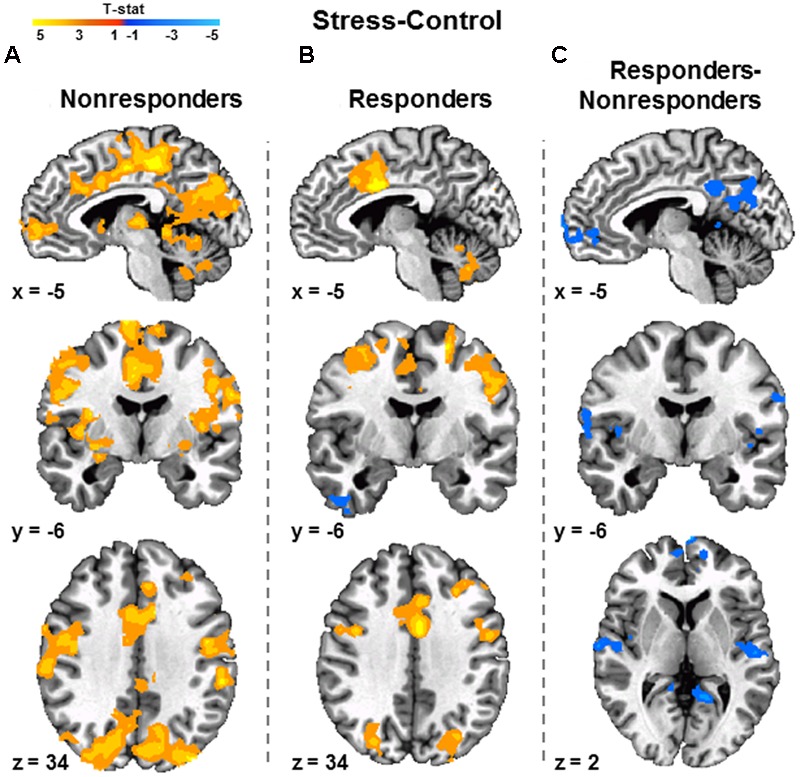
**Relationship between cortisol release and fMRI signal during the MIST.** Differential activity (Stress vs. Control) associated with cortisol Non-responders (*n* = 30) **(A)** and Responders (*n* = 22) **(B)**. A two-sample *t*-test revealed Non-responders demonstrated greater differential ventromedial prefrontal cortex and posterior cingulate cortex activity than Responders **(C)**. Images are FWE corrected at *p* < 0.05 and presented in radiologic view.

Functional MRI data for the Stress vs. Control contrast were compared between cortisol Responders and Non-responders. Cortisol Non-responders demonstrated greater differential activity than Responders within several brain regions including the vmPFC, posterior cingulate cortex (PCC), insula, and superior temporal gyrus (**Figure 4**; **Supplementary Table S2**). Prior research reported amygdala activity associated with cortisol reactivity as a continuous measure (Henckens et al., 2016). We therefore assessed the relationship between brain activity and cortisol reactivity using cortisol response as a continuous variable with a region of interest mask including the PFC, amygdala, hippocampus, and hypothalamus. The results from this analysis revealed vmPFC activation patterns similar to that observed in the Responder vs. Non-responder analysis (**Supplementary Figure S2**), but no activity was observed in the other ROIs.

### Brain and Behavior Results

**FIGURE 5:**
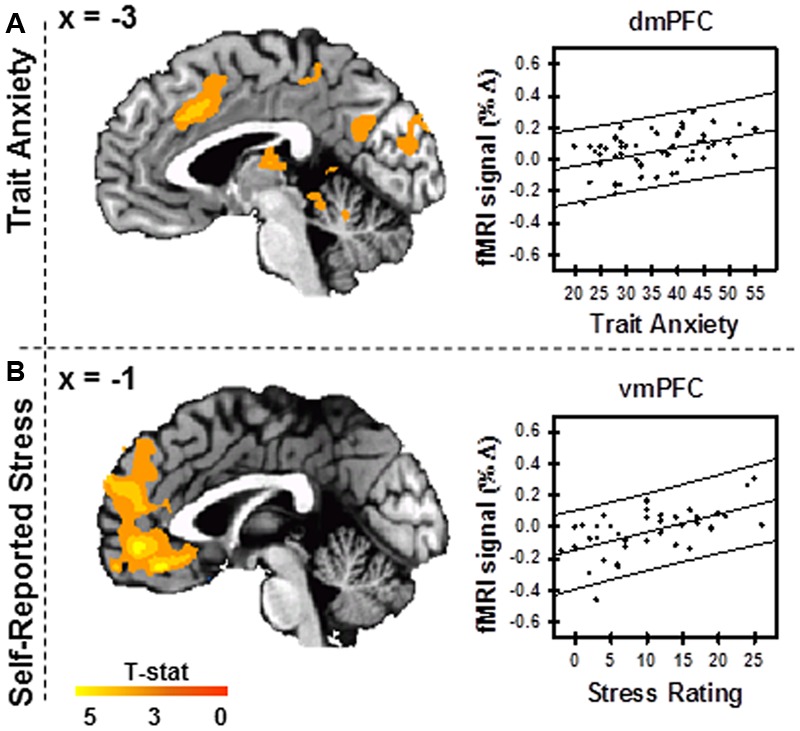
**Trait anxiety and self-reported stress.** Variance in BOLD fMRI signal during Stress vs. Control conditions varied with trait anxiety score **(A)** (*N* = 53) and self-reported stress **(B)** (*n* = 40). Scatterplots display peak activation within the dmPFC (*x* = -3, *y* = 19, *z* = 35) **(A)** and vmPFC (*x* = -1, *y* = -45, *z* = -16) **(B)**. Images are FWE corrected at *p* < 0.05 and presented in radiologic view.

Variance in BOLD signal during Stress vs. Control MIST was positively correlated with trait anxiety within the dmPFC, PCC, and insula (**Figure 5A**; **Supplementary Table S3**). Variance in BOLD signal during Stress vs. Control MIST was positively correlated with self-reported stress within the PFC with peak coordinates in the vmPFC (**Figure 5B**; **Supplementary Table S4**).

### Conjunction Analysis Results

**FIGURE 6:**
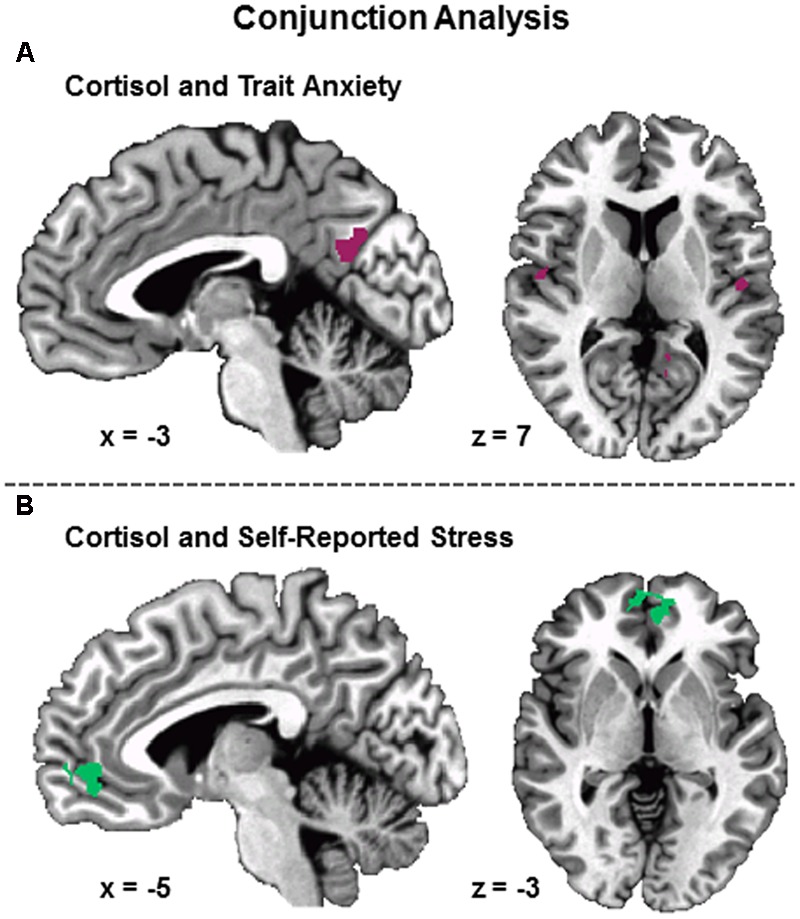
**Conjunction of psychobiological measures associated with differential (Stress vs. Control) BOLD fMRI signal during the MIST.** Conjunction of cortisol and trait anxiety showed overlapping areas of activation within posterior cingulate cortex and bilateral superior temporal gyrus **(A)**. Conjunction of self-reported stress and cortisol showed overlapping areas of activation within ventromedial prefrontal cortex **(B)**. No overlapping areas were identified between trait anxiety and self-reported stress or the conjunction of trait anxiety, self-reported stress, and cortisol. Results FWE corrected at *p* < 0.05.

The conjunction analysis revealed a common region of differential activity between cortisol reactivity and self-reported stress within the vmPFC (**Figure 6**). The conjunction analysis also revealed common regions of differential activity between cortisol reactivity and trait anxiety within the PCC and insula. No regions of common activation were found between self-reported stress and trait anxiety, and no regions were found to be common among all three measures.

## Discussion

Although the acute stress response is an adaptive, allostatic process under normal conditions, chronic stress can lead to or exacerbate health-related problems including depression, anxiety, and addiction-related behaviors (Brydges et al., 2014). Although it is widely assumed a variety of factors impact stress susceptibility, we do not fully understand the neural processes that lead to susceptibility in one individual but not in another. In this study we investigated the neural correlates of the stress response to better understand how differences in stress-related behavior vary with differences in brain function. We found less stress-induced activity within the vmPFC for cortisol Responders than Non-responders, consistent with the view that this region of the brain regulates the emotional response to stress. Further, the neural response to stress within the dmPFC and vmPFC varied with individual differences in self-reported stress and trait anxiety. These findings suggest the PFC regulates emotion and influences individual differences in stress reactivity.

### Cortisol Reactivity

Considerable heterogeneity was observed in the cortisol response within our sample. Forty percent of individuals in this study demonstrated an increase in salivary cortisol levels in response to stress and were identified as cortisol Responders. This is consistent with prior work assessing the cortisol response in the MRI environment (Pruessner et al., 2008; Dedovic et al., 2009b). While the BOLD response measured during the rapid event-related design of the MIST is temporally short in comparison to the long acting effects of cortisol, phasic responses in brain activity mediate the interpretation and psychological response to the MIST and influence subsequent HPA axis reactivity. The correlates of these phasic responses can be used to infer brain activation associated with cortisol reactivity. In the present study, cortisol Non-responders demonstrated greater differential activity within the vmPFC than cortisol Responders. This finding is consistent with the view that vmPFC regulates amygdala activity (Motzkin et al., 2015) and influences the cortisol response to stress (Pruessner et al., 2008). More specifically, the PFC and hippocampus are thought to play an inhibitory role in cortisol release via connections to the hypothalamus while the amygdala is thought to play an excitatory role (Ulrich-Lai and Herman, 2009). However, amygdala and hippocampal activity did not vary with cortisol release in the present study. Of particular and novel interest, cortisol reactivity in the present study was associated with both vmPFC and PCC activity, regions which are commonly included within the default mode network (Laird et al., 2009). This finding suggests that connectivity within this network may influence cortisol reactivity to acute stress. Prior research has demonstrated a relationship between this network and intersubject variability in perceived stress scale score (Soares et al., 2016). Future studies should examine the association between stress reactivity and brain connectivity within the vmPFC-PCC network.

### Trait Anxiety

The relationship between trait anxiety and differential (Stress – Control) neural activity was also examined in the present study. Findings from the regression analysis demonstrated that differential dmPFC activity increased as trait anxiety increased. Prior work has demonstrated an association between trait anxiety and dmPFC activity during Pavlovian conditioning (Wood et al., 2012). In addition, perceived anxiety has been associated with dmPFC activity during a stressful task (Wang et al., 2005). The association between trait anxiety and dmPFC activation in the present study is consistent with results from prior studies (Wang et al., 2005; Wood et al., 2012). Also consistent with prior research, we did not observe any relationship between trait anxiety and cortisol reactivity in the present paper (Bohen et al., 1991; Vedhara et al., 2003; Takai et al., 2004). Prior research indicates that basal cortisol correlates with trait anxiety scores, but stress reactivity to an acute stressor does not (Takahashi et al., 2005). Other meta analyses suggest that there is no measurable relationship between personality factors and cortisol release (Kudielka et al., 2009; Campbell and Ehlert, 2012). While anxiety and cortisol reactivity did not correlate, trait anxiety and cortisol reactivity co-localized within regions of the insula and PCC (**Figure 6A**). The commonality in brain regions underlying trait anxiety and stress reactivity may suggest a common neural mechanism by which personality traits are integrated into physiological output.

### Self-Reported Stress

The present study also investigated the relationship between self-reported stress and differential brain activity in response to stress. The regression analysis demonstrated that differential vmPFC activity increased as self-reported stress increased. Prior research suggests that perceived stress is associated with vmPFC activity during stress (Wang et al., 2005). More specifically, prior research suggests that individuals reporting high stress have greater differential (Stress vs. Control) subgenual and anterior cingulate activity to the MIST than individuals reporting low stress (Albert et al., 2015). No association was observed between cortisol response and self-reported stress, consistent with prior research (Albert et al., 2015). However, in the present study self-reported stress co-localized with activity associated with cortisol reactivity. The similarities in localization of function between self-reported stress and cortisol reactivity (**Figure 6B**) suggest the vmPFC integrates perceptions of stress and emotional reactivity in response to acute stress. However, self-reported stress did not correlate with cortisol reactivity in the present study, suggesting participants with high HPA axis arousal may either be unaware or unable to interpret stressful internal physiological states.

### Additional Physiological Measures

Both HR and SCR demonstrated a clear stress response from Control to Stress MIST in the present study. These findings suggest the inclusion of HR and SCR in stress studies may provide improved temporal resolution in measurements of the stress response over the slowly evolving fluctuations in endocrine mediated hormone levels. SCR and HR have the additional benefit of being measured continuously during task, while cortisol and other endocrine measures can only be measured at discrete time points non-concurrent with the stress task. This suggests a need for multimodal assessments of the emotional response during fMRI studies of stress. Future stress research may benefit from assessing SCR and HR in addition to cortisol.

### Limitations

There are several limitations to this study. First, while inter-subject variability was observed in the cortisol response to stress, we did not observe an increase in cortisol level following the stress task for the group. Several other studies have also failed to find increased cortisol following stress tasks completed in the scanning environment (Dedovic et al., 2009b; Allendorfer et al., 2014). This may be due to an elevated baseline in pre-scanning cortisol levels due to anticipation of a medical scanning procedure (Tessner et al., 2006; Muehlhan et al., 2011). Another aspect of the study that may have impacted cortisol levels was variability in the time of day that the study was completed. Cortisol fluctuates diurnally, with the lowest levels of basal cortisol observed in the afternoon. While we attempted to account for diurnal fluctuations in cortisol by including the time of day cortisol was sampled in our regression analysis, it is still possible that these diurnal rhythms may have impacted the cortisol reactivity of our participants. Further, the present experimental design employed a Control scan that always preceded the Stress scan. While the present study avoided any impact of the stress response on the baseline control scan measurement, it is still possible that other order effects may impact the present data. Finally, several important updates to AFNI scripts used to control FWE were implemented after this study was conducted and the paper prepared. The present paper controlled FWE according to the standards available at the time. It is possible that the smaller clusters reported in the Supplemental Tables may not survive using newer cluster correction software and should thus be interpreted with some caution.

## Conclusion

We observed differences in stress evoked activation in the PFC during psychosocial stress. Cortisol Responders demonstrated less vmPFC activity than Non-responders. Prior work has linked diminished vmPFC activity to reductions in regulatory control over emotion (Ochsner et al., 2002, 2012; Delgado et al., 2008; Beauchamp et al., 2016). Furthermore, trait anxiety and self-reported stress were positively associated with the PFC response to stress. Though cortisol reactivity and self-reported stress were not correlated, the vmPFC appears to influence both of these stress-related measures. Taken together these findings suggest a multifaceted role for the vmPFC that integrates self-reported experiences and the endocrine response to stress. Finally, these findings suggest a potential neural mechanism that influences individual differences in stress reactivity.

## Author Contributions

MW: Data collection, fMRI analysis, interpretation, drafting, and revising article. NH: Data collection, HR analysis, and revising article. KW: Data collection, revising article. TO: SCR and HR analysis, revising article. DG: cortisol analysis, revising article. SM: Study design, revising article. DK: Study design, interpretation, drafting and revising article.

## Conflict of Interest Statement

In the interest of full disclosure DG is Founder and Chief Scientific and Strategy Advisor at Salimetrics LLC and SalivaBio LLC and these relationships are managed by the policies of the committees on conflict of interest at the Johns Hopkins University School of Medicine and the University of California at Irvine. All the other authors declare that the research was conducted in the absence of any commercial or financial relationships that could be construed as a potential conflict of interest.

## Supplementary Material

FIGURE S1 **Montreal Imaging Stress Task (MIST).** Each trial (6 s duration) was separated by a 1.0–3.0 s inter-trial interval **(A)**, followed by a math problem presented for 0.5–5.0 s **(B)**. Participants used an MRI compatible joystick to highlight their math answer (in yellow) and a button on the joystick to make their selection. Once the participant responded, a fixation cross appeared for the remainder of the 5.0 s **(C)**. A fixation cross always appeared for at least 0.5 s following the math problem. Following the fixation cross participants were given visual feedback (0.5 s duration) **(D)**.

FIGURE S2 **Cortisol regressed on differential brain activation (Stress – Control).** Brain data were masked to include regions of interest: ventromedial, ventrolateral, dorsomedial, dorsolateral prefrontal cortex (PFC), amygdala, hippocampus, and hypothalamus. Cortisol reactivity scores were regressed as a continuous variable onto regions of interest and corrected for FWE (*p* < 0.05). This regression revealed similar patterns of activation within the ventromedial PFC to that of the cortisol Responder vs. Non-responder analysis.

TABLE S1 **Brain activation for Stress-Control conditions**.

TABLE S2 **Brain activation coordinates for cortisol Responders vs. Non-responders**.

TABLE S3 **Brain activation coordinates for trait anxiety scale regression**.

TABLE S4 **Brain activation coordinates for self-reported stress regression**.
