# Supplementary figures and images for: Prefrontal Cortex Activity Is Associated with Biobehavioral Components of the Stress Response
Source: Front Hum Neurosci. 2016 Nov 17;10:583. doi: 10.3389/fnhum.2016.00583 (PMC5112266; doi:10.3389/fnhum.2016.00583)

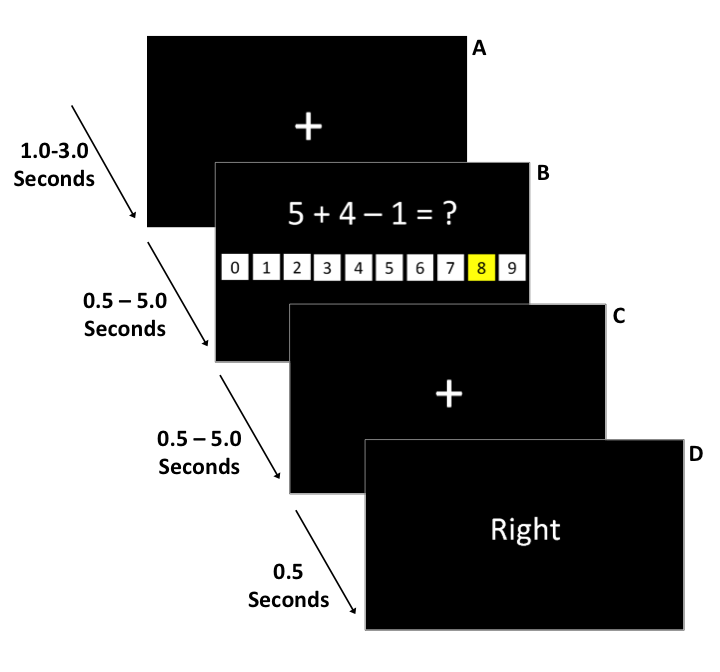

Supplement: FIGURE S1 — Montreal Imaging Stress Task (MIST). Each trial (6 s duration) was separated by a 1.0–3.0 s inter-trial interval (A), followed by a math problem presented for 0.5–5.0 s (B). Participants used an MRI compatible joystick to highlight their math answer (in yellow) and a button on the joystick to make their selection. Once the participant responded, a fixation cross appeared for the remainder of the 5.0 s (C). A fixation cross always appeared for at least 0.5 s following the math problem. Following the fixation cross participants were given visual feedback (0.5 s duration) (D). [file Image_1.TIFF]

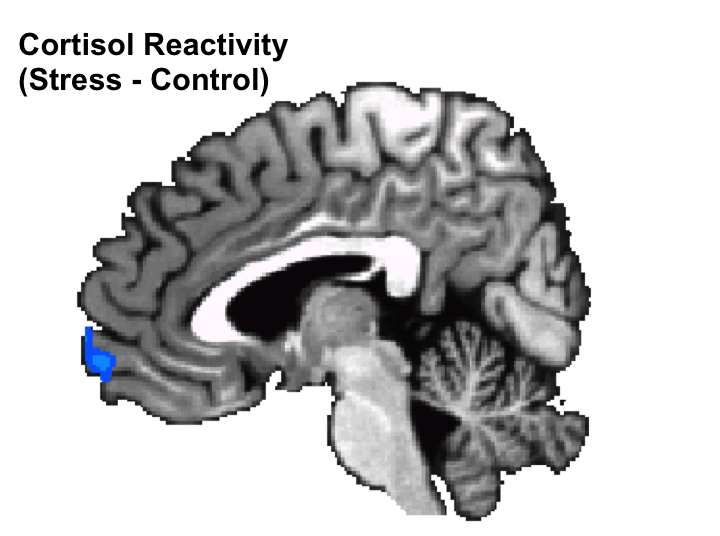

Supplement: FIGURE S2 — Cortisol regressed on differential brain activation (Stress – Control). Brain data were masked to include regions of interest: ventromedial, ventrolateral, dorsomedial, dorsolateral prefrontal cortex (PFC), amygdala, hippocampus, and hypothalamus. Cortisol reactivity scores were regressed as a continuous variable onto regions of interest and corrected for FWE (p < 0.05). This regression revealed similar patterns of activation within the ventromedial PFC to that of the cortisol Responder vs. Non-responder analysis. [file Image_2.TIFF]
