# Supplementary material for: Prefrontal Cortex Activity Is Associated with Biobehavioral Components of the Stress Response
Source: Front Hum Neurosci. 2016 Nov 17;10:583. doi: 10.3389/fnhum.2016.00583 (PMC5112266; doi:10.3389/fnhum.2016.00583)
Supplement: TABLE S1 — Brain activation for Stress-Control conditions. [file Table_1.DOCX]

**Supplemental Table S1**. Brain activation for Stress-Control Conditions

| **Region** | **Hemisphere** | **x,y,z** | **Peak *t* Value** |
| --- | --- | --- | --- |
| **Math Response** |  |  |  |
| Cluster 1 (60621 voxels) |  |  |  |
| *Cluster Sub Regions* |  |  |  |
| Precentral | R | 40,-18,56 | 7.29 |
| Precentral | L | -22,-24,64 | 6.92 |
| Postcentral | R | 30,-28,60 | 6.49 |
| SMA | R | 6,-22,56 | 6.04 |
| SMA | L | -10,-20,50 | 5.17 |
| Paracentral Lobule | R | 10,-40,58 | 5.26 |
| Paracentral Lobule | L | -6,-20,56 | 3.78 |
| Middle Cingulum | L | -14,-40,54 | 5.37 |
| Middle Cingulum | R | 14,-10,42 | 5.01 |
| Precuneus | R | 14,-46,60 | 5.36 |
| Precuneus | L | -12,-50,64 | 3.36 |
| Anterior Cingulum | L | -4,8,30 | 5.60 |
| Superior Frontal | R | 14,-12,76 | 4.75 |
| Opercular Rolandic | R | 50,4,2 | 4.20 |
| Superior Parietal | R | 16,-46,66 | 6.15 |
| Superior Parietal | L | -18,-40,62 | 3.41 |
| Cluster 2 (19514 voxels) |  |  |  |
| *Cluster Sub Regions* |  |  |  |
| Middle Occipital | L | -50,-78,6 | 5.04 |
| Calcarine | L | -12,-60,12 | 4.16 |
| Superior Occipital | L | -14,-82,26 | 7.03 |
| Cuneus | L | -16,-60,20 | 4.37 |
| Fusiform | L | -28,-54,-12 | 5.05 |
| Cerebelum 4,5 | L | -12,-52,-14 | 4.17 |
| Vermis 6 |  | 0,-64,-18 | 3.51 |
| Vermis 4,5 |  | -2,-62,-12 | 3.59 |
| Lingual | L | -20,-48,-6 | 4.21 |
| Precuneus | L | -14,-56,18 | 4.63 |
| Middle Temporal | L | -46,-60,-4 | 4.61 |
| Inferior Occipital | L | -36,-66,-6 | 3.59 |
| Inferior Parietal | L | -30,-70,44 | 3.70 |
| Superior Parietal | L | -18,-64,44 | 3.00 |
| Cluster 3 (4528 voxels) |  |  |  |
| *Cluster Sub Regions* |  |  |  |
| Putamen | L | -16,6,-6 | 6.43 |
| Pallidum | L | -26,-12,-4 | 4.55 |
| Caudate | L | -14,-16,0 | 4.03 |
| Thalamus | L | -22,-18,6 | 4.38 |
| Cluster 4 (8768 voxels) |  |  |  |
| *Cluster Sub Regions* |  |  |  |
| Putamen | R | 20,10,-8 | 5.91 |
| Insula | R | 34,-12,18 | 4.36 |
| Caudate | R | 8,8,0 | 5.46 |
| Pallidum | R | 12,2,-4 | 4.89 |
| Inferior Tri Frontal | R | 44,24,10 | 3.64 |
| Amygdala | R | 30,-6,-12 | 3.93 |
| Opercular Rolandic | R | 38,-20,20 | 3.36 |
| Inferior OFC | R | 36,34,-4 | 3.70 |
| Cluster 5 (17500) |  |  |  |
| *Cluster Sub Regions* |  |  |  |
| Middle Occipital | R | 44,-72,4 | 4.44 |
| Superior Occipital | R | 28,-78,32 | 5.20 |
| Middle Temporal | R | 46,-58,4 | 5.17 |
| Thalamus | L | -8,28,0 | 4.89 |
| Precuneus | R | 20,-58,22 | 4.50 |
| Lingual Gyrus | R | 10,-32,-8 | 4.11 |
| Calcarine | R | 12,-60,18 | 4.59 |
| Fusiform | R | 32,-50,-10 | 4.05 |
| Cuneus | R | 16,-72,36 | 4.03 |
| Thalamus | R | 10,-26,-2 | 4.06 |
| Hippocampus | L | -14,-36,0 | 4.95 |
| Hippocampus | R | 30,-64,-6 | 3.97 |
| Inferior Temporal | R | 44,-56,-6 | 4.00 |
| Parahippocampal | R | 18,-36,-6 | 4.16 |
| Lingual | L | -8,-34,-2 | 4.28 |
| Cluster 6 (1487 voxels) |  |  |  |
| *Cluster Sub Regions* |  |  |  |
| Middle Frontal | L | -42,38,16 | 5.09 |
| Inferior Tri Frontal | L | -38,34,32 | 4.68 |
| Cluster 7 (1100 voxels) |  |  |  |
| *Cluster Sub Regions* |  |  |  |
| Middle Frontal | L | -42,38,16 | 5.09 |
| Cluster 8 (4715 voxels) |  |  |  |
| *Cluster Sub Regions* |  |  |  |
| Precentral | L | -58,-2,30 | 4.57 |
| Postcentral | L | -46,-10,42 | 4.90 |
| Cluster 9 (919 voxels) |  |  |  |
| *Cluster Sub Regions* |  |  |  |
| Supramarginal Gyrus | R | 62,-20,32 | 4.80 |
| Cluster 10 (2793 voxels) |  |  |  |
| *Cluster Sub Regions* |  |  |  |
| Vermis 8 |  | 4,-68,-32 | 4.67 |
| Vermis 9 |  | 2,-52,-38 | 3.62 |
| Cerebelum 8 | R | 8,-66,40 | 3.30 |
| Cluster 11 (1100 voxels) |  |  |  |
| *Cluster Sub Regions* |  |  |  |
| Superior Frontal | L | -22,14,50 | 4.39 |
| Cluster 12 (1927 voxels) |  |  |  |
| *Cluster Sub Regions* |  |  |  |
| Insula | L | -30,18,10 | 4.30 |
| Caudate | L | -10,-4,14 | -5.11 |

Uncorrected *p*<0.005, cluster correction of 622 voxels yields a corrected *p* of <0.05 and a minimum T-value of 2.93.
