# Supplementary material for: Prefrontal Cortex Activity Is Associated with Biobehavioral Components of the Stress Response
Source: Front Hum Neurosci. 2016 Nov 17;10:583. doi: 10.3389/fnhum.2016.00583 (PMC5112266; doi:10.3389/fnhum.2016.00583)
Supplement: TABLE S2 — Brain activation coordinates for cortisol Responders vs. Non-responders. [file Table_2.DOCX]

**Supplemental Table S2**. Brain activation coordinates for cortisol Responders vs Nonresponders.

| **Region** | **Hemisphere** | **x,y,z** | **Peak *t* Value** |
| --- | --- | --- | --- |
| **Math Response** |  |  |  |
| Cluster 1 (5941 voxels) |  |  |  |
| *Cluster Sub Regions* |  |  |  |
| Medial OFC | L | -8,62,-8 | 3.70 |
| Medial OFC | R | 6,62,-10 | 4.15 |
| Middle OFC | L | -26,50,-14 | 2.97 |
| Superior Medial Frontal | L | -4,72,2 | 3.46 |
| Anterior Cingulum | L | -6,48,-2 | 3.20 |
| Superior Medial Frontal | R | 0,68,2 | 2.86 |
| Superior OFC | R | 12,64,-18 | 2.87 |
| Superior Frontal | L | -8,72,8 | 3.13 |
| Cluster 2 (13731 voxels) |  |  |  |
| *Cluster Sub Regions* |  |  |  |
| Precuneus | R | 20,-50,-22 | 4.05 |
| Precuneus | L | -12,-58,14 | 3.51 |
| Cuneus | L | 0,-72,30 | 3.28 |
| Posterior Cingulum | L | -10,-48,30 | 3.04 |
| Calcarine | L | -6,-48,4 | 2.55 |
| Middle Cingulum | L | -14,-50,32 | 3.59 |
| Posterior Cingulum | R | 6,-42,14 | 2.22 |
| Middle Cingulum | R | 6,-50,32 | 3.86 |
| Lingual | L | -10,-38,-6 | 2.07 |
| Cuneus | R | 4,-84,30 | 2.30 |
| Cerebelum 4,5 | L | -8,-42,-6 | 2.14 |
| Lingual | R | -12,-50,2 | 3.32 |
| Cluster 3 (4360 voxels) |  |  |  |
| *Cluster Sub Regions* |  |  |  |
| Superior Temporal Pole | R | 48,14,-14 | 3.86 |
| Insula | R | 44,4,-12 | 3.62 |
| Middle Temporal Pole | R | 58,8,-20 | 3.14 |
| Inferior OFC | R | 30,24,-26 | 3.12 |
| Superior Temporal | R | 54,12,-8 | 2.69 |
| Middle Temporal | R | 60,4,-18 | 3.10 |
| Cluster 4 (3616 voxels) |  |  |  |
| *Cluster Sub Regions* |  |  |  |
| Superior Temporal | R | 68,-14,0 | 3.34 |
| Postcentral | R | 64,-4,16 | 3.18 |
| Opercular Rolandic | R | 64,-6,10 | 2.58 |
| Heschl’s Gyrus | R | 58,-10,6 | 2.22 |
| Insula | R | 44,-6,6 | 2.47 |
| Middle Temporal | R | 54,-14,-10 | 2.27 |
| Cluster 5 (3427 voxels) |  |  |  |
| *Cluster Sub Regions* |  |  |  |
| Superior Temporal | L | -52,-16,4 | 3.05 |
| Postcentral | L | -56,-10,16 | 2.78 |
| Insula | L | -42,-10,0 | 2.49 |
| Middle Temporal | L | -64,-28,2 | 2.37 |
| Opercular Rolandic | L | -48,-10,18 | 2.42 |

Uncorrected *p*<0.05, cluster correction of 3000 voxels yields a corrected *p* of <0.05 and a minimum T-value of 2.01.
