# Supplementary material for: Prefrontal Cortex Activity Is Associated with Biobehavioral Components of the Stress Response
Source: Front Hum Neurosci. 2016 Nov 17;10:583. doi: 10.3389/fnhum.2016.00583 (PMC5112266; doi:10.3389/fnhum.2016.00583)
Supplement: TABLE S3 — Brain activation coordinates for trait anxiety scale regression. [file Table_3.docx]

**Supplemental Table S3**. Brain activation coordinates for trait anxiety scale regression.

| **Region** | **Hemisphere** | **x,y,z** | **Peak *t* Value** |
| --- | --- | --- | --- |
| **Math Response** |  |  |  |
| Cluster 1 (24581 voxels) |  |  |  |
| *Cluster Sub Regions* |  |  |  |
| Thalamus | R | 18,-30,4 | 3.99 |
| Thalamus | L | -22,-28,4 | 3.91 |
| Hippocampus | L | -32,-28,-10 | 4.83 |
| Precuneus | L | -12,-38,2 | 3.19 |
| Calcarine | L | -18,-58,10 | 4.19 |
| Lingual | L | -30,-46,-6 | 3.06 |
| Cerebelum 4,5 | L | -8,-54,-22 | 2.84 |
| Fusiform | L | -32,-56,-10 | 2.95 |
| Fusiform | R | 28,-36,-16 | 3.00 |
| Parahippocampal | L | -24,-42,-4 | 3.85 |
| Parahippocampal | R | 26,-28,-14 | 3.37 |
| Caudate | L | -10,0,12 | 2.96 |
| Cuneus | L | -6,-64,24 | 2.65 |
| Inferior Temporal | L | -38,-30,-24 | 3.27 |
| Posterior Cingulum | L | -10,-44,8 | 3.16 |
| Cerebelum 3 | L | -4,-38,-8 | 2.53 |
| Vermis 4,5 |  | 2,-54,-16 | 2.36 |
| Inferior Occipital | L | -40,-66,-6 | 3.21 |
| Lingual | R | 14,-34,-2 | 2.06 |
| Cluster 2 (4724 voxels) |  |  |  |
| *Cluster Sub Regions* |  |  |  |
| Superior Temporal | R | 52,-36,12 | 4.01 |
| Heschl’s Gyrus | R | 46,-18,8 | 2.91 |
| Opercular Rolandic | R | 46,-28,18 | 2.77 |
| Cluster 3 (5797 voxels) |  |  |  |
| *Cluster Sub Regions* |  |  |  |
| Cuneus | L | -10,-86,30 | 3.52 |
| Middle Occipital | L | -38,-90,8 | 3.99 |
| Superior Occipital | L | -24,-96,22 | 3.37 |
| Cluster 4 (4471 voxels) |  |  |  |
| *Cluster Sub Regions* |  |  |  |
| Middle Cingulum | L | -10,-36,52 | 3.16 |
| Middle Cingulum | R | 18,-32,42 | 2.53 |
| Precuneus | L | -14,-58,48 | 2.83 |
| Precuneus | R | 2,-42,58 | 2.29 |
| Paracentral Lobule | L | -2,-34,50 | 2.44 |
| Paracentral Lobule | R | 12,38,54 | 3.93 |
| Superior Parietal | L | -20,-54,-44 | 2.83 |
| Postcentral | R | 16,-30,60 | 2.72 |
| Cluster 5 (3532 voxels) |  |  |  |
| *Cluster Sub Regions* |  |  |  |
| Calcarine | R | 16,-60,10 | 3.88 |
| Precuneus | R | 14,-64,30 | 2.76 |
| Lingual | R | 20,-64,-2 | 2.51 |
| Fusiform | R | 24,-66,-8 | 2.69 |
| Superior Occipital | R | 28,-64,36 | 2.41 |
| Middle Occipital | R | 32,-64,36 | 2.91 |
| Cluster 6 (7488 voxels) |  |  |  |
| *Cluster Sub Regions* |  |  |  |
| Middle Cingulum | L | -2,20,36 | 3.76 |
| Middle Cingulum | R | 8,4,30 | 3.39 |
| Superior Frontal | R | 16,0,54 | 3.23 |
| SMA | L | -2,14,46 | 2.56 |
| SMA | R | 16,2,66 | 2.86 |
| Superior Medial Frontal | L | -8,20,42 | 2.09 |
| Anterior Cingulum | R | 4,28,26 | 2.58 |
| Cluster 7 (3190 voxels) |  |  |  |
| Superior Temporal Pole | R | 58,20,-14 | 3.44 |
| Opercular Rolandic | R | 54,-4,8 | 2.91 |
| Middle Temporal | R | 64,4,-28 | 3.46 |
| Middle Temporal Pole | R | 66,6,-20 | 3.29 |
| Inferior OFC | R | 60,22,-4 | 2.35 |
| Cluster 8 (4470 voxels) |  |  |  |
| Superior Temporal | L | -60,-40,16 | 3.30 |
| Supramarginal Gyrus | L | -44,-46,28 | 3.08 |
| Heschl’s Gyrus | L | -32,-26,6 | 2.47 |
| Middle Temporal | L | -70,-28,4 | 2.39 |
| Oper Rolandic | L | -40,-20,12 | 2.42 |
| Postcentral | L | -60,-20,14 | 2.83 |
| Angular | L | -42,-50,28 | 2.99 |
| Cluster 9 (3668 voxels) |  |  |  |
| Putamen | R | 24,10,10 | 3.18 |
| Pallidum | R | 22,-2,-2 | 3.28 |
| Caudate | R | 22,18,6 | 2.94 |
| Insula | R | 36,8,6 | 2.27 |

Uncorrected *p*<0.05, cluster correction of 3000 voxels yields a corrected *p* of <0.05 and a minimum T-value of 2.01.
