# Supplementary material for: Prefrontal Cortex Activity Is Associated with Biobehavioral Components of the Stress Response
Source: Front Hum Neurosci. 2016 Nov 17;10:583. doi: 10.3389/fnhum.2016.00583 (PMC5112266; doi:10.3389/fnhum.2016.00583)
Supplement: TABLE S4 — Brain activation coordinates for self-reported stress regression. [file Table_4.DOCX]

**Supplemental Table S4**. Brain activation coordinates for self-reported stress regression.

| **Region** | **Hemisphere** | **x,y,z** | **Peak *t* Value** |
| --- | --- | --- | --- |
| **Math Response** |  |  |  |
| Cluster 1 (42536 voxels) |  |  |  |
| *Cluster Sub Regions* |  |  |  |
| Superior Medial Frontal | L | -10,52,12 | 4.17 |
| Superior Medial Frontal | R | 14,56,42 | 3.25 |
| Anterior Cingulum | L | -8,38,-6 | 3.35 |
| Anterior Cingulum | R | 14,34,6 | 3.33 |
| Inferior OFC | L | -36,26,-6 | 4.26 |
| Inferior OFC | R | 26,28,-24 | 2.94 |
| Inferior Tri Frontal | L | -54,26,6 | 4.41 |
| Medial OFC | L | 0,52,-6 | 4.32 |
| Medial OFC | R | 8,34,-10 | 4.10 |
| Middle Temporal | L | -62,-24,-10 | 3.94 |
| Superior Temporal Pole | L | -48,12,-16 | 3.22 |
| Superior Temporal Pole | R | 24,6,-22 | 2.58 |
| Superior Frontal | L | -32,58,0 | 3.66 |
| Superior Frontal | R | 20,56,40 | 3.67 |
| Superior OFC | L | -12,22,-18 | 2.57 |
| Superior OFC | R | 20,14,-20 | 2.54 |
| Rectus | L | 0,40,-16 | 4.72 |
| Rectus | R | 6,22,-22 | 3.34 |
| Insula | L | -26,18,-14 | 3.86 |
| Inferior Oper Frontal | L | -16,14,12 | 3.46 |
| Superior Temporal | L | -54,-10,0 | 3.42 |
| Olfactory | R | 6,20,-8 | 3.75 |
| Middle Frontal | L | -24,54,14 | 2.16 |
| Middle Temporal Pole | L | -50,10,-26 | 2.92 |
| Middle OFC | R | 24,38,-22 | 2.65 |
| Postcentral | L | -62,2,16 | 2.35 |
| Cluster 2 (3026 voxels) |  |  |  |
| *Cluster Sub Regions* |  |  |  |
| Middle Temporal Pole | R | 40,20,-36 | 3.50 |
| Superior Temporal Pole | R | 38,18,-26 | 3.71 |
| Inferior OFC | R | 28,16,-22 | 2.64 |
| Parahippocampal | R | 22,16,-32 | 2.50 |
| Cluster 3 (3111 voxels) |  |  |  |
| *Cluster Sub Regions* |  |  |  |
| Inferior OFC | R | 44,34,-20 | 3.19 |
| Inferior Triangular Gyrus | R | 50,32,2 | 3.54 |
| Middle OFC | R | 40,40,-16 | 3.66 |

Uncorrected *p*<0.05, cluster correction of 3000 voxels yields a corrected *p* of <0.05 and a minimum T-value of 2.01.
